# Development of a 1550-nm InAs/GaAs Quantum Dot Saturable Absorber Mirror with a Short-Period Superlattice Capping Structure Towards Femtosecond Fiber Laser Applications

**DOI:** 10.1186/s11671-019-3188-3

**Published:** 2019-12-02

**Authors:** Cheng Jiang, Jiqiang Ning, Xiaohui Li, Xu Wang, Ziyang Zhang

**Affiliations:** 1 Key Lab of Nanodevices and Applications, Suzhou Institute of Nano-Tech and Nano-Bionics, Chinese Academy of Sciences, Suzhou, 215123 China; 2 Nano Science and Technology Institute, University of Science and Technology of China, Suzhou, 215123 China; 3 Vacuum Interconnected Nanotech Workstation, Suzhou Institute of Nano-Tech and Nano-Bionics, Chinese Academy of Sciences, Suzhou, 215123 China; 4 School of Physics & Information Technology, Shaanxi Normal University, Xi’an, China

**Keywords:** InAs/GaAs QDs, Short-period superlattice, Phonon bottleneck effect, SESAMs, Mode-locked lasers

## Abstract

Low-dimensional III–V InAs/GaAs quantum dots (QDs) have been successfully applied to semiconductor saturable absorber mirrors (SESAMs) working at a 900–1310-nm wavelength range for ultrafast pulsed laser applications benefitting from their broad bandwidth, wavelength flexibility, and low saturation fluence. However, it is very challenging to obtain a high-performance QD-SESAM working at the longer wavelength range around 1550 nm due to the huge obstacle to epitaxy growth of the QD structures. In this work, for the first time, it is revealed that, the InAs/GaAs QD system designed for the 1550-nm light emission range, the very weak carrier relaxation process from the capping layers (CLs) to QDs is mainly responsible for the poor emission performance, according to which we have developed a short-period superlattice (In_0.20_Ga_0.80_As/In_0.30_Ga_0.70_As)_5_ as the CL for the QDs and has realized ~ 10 times stronger emission at 1550 nm compared with the conventional InGaAs CL. Based on the developed QD structure, high-performance QD-SESAMs have been successfully achieved, exhibiting a very small saturation intensity of 13.7 MW/cm^2^ and a large nonlinear modulation depth of 1.6 %, simultaneously, which enables the construction of a 1550-nm femtosecond mode-locked fiber lasers with excellent long-term working stability.

## Introduction

1550-nm mode-locked femtosecond pulsed lasers have wide applications in optical communication, ultrafast optics, and non-linear optics due to their high peak power, low thermal effect, and high pulse energy [1–5]. The saturable absorber (SA) with the wide optical bandwidth, fast response time and low loss properties are the critical optical component for such ultrashort pulsed lasers [6–9]. In addition, high damage threshold of the SA is highly desirable for long-term stable operation of a mode-locked laser [10–13]. Recently, two-dimensional (2D) materials such as graphene, topological insulators, black phosphorus and transition metal dichalcogenides have attracted lots of attention for their application as SAs for mode-locked femtosecond pulsed lasers [14–21]. However, their low damage threshold and poor working stability have severely hindered their wide applications [22, 23]. Quantum well (QW)-based SESAMs are regarded as a commercial candidate for mode-locked ultrafast lasers due to their high repeatability and excellent operation stability, but the narrow operation bandwidths and small modulation depth are still the huge barriers to the realization of femtosecond ultrashort pulses [24].

Recently, featured with board operation bandwidth and fast carrier recovery time [25–31], self-assembled InAs quantum dots (QDs) grown via the Stranski-Krastanow mode have emerged as an excellent choice for SESAMs to construct mode-locked pulsed lasers. To achieve the working wavelength around 1550 nm, InP based InGaAsP QWs are usually employed. The bandgaps of GaAs based InGaAs QDs can be generally engineered to cover the spectral range from 980 to 1310 nm, and a longer wavelength beyond 1310 nm requires much higher indium content in the QD capping layers (CLs). Quaternary InGaAsSb (InGaNAs) alloys and very high In % (> 30 %) InGaAs CLs have been employed to engineer the QD bandgap towards the long wavelength of 1550 nm [32, 33]. However, the quaternary alloy CLs significantly complicate the epitaxial growth process, and the high In content in InGaAs CLs degrades the crystalline and optical quality of the QDs, which introduces more nonradiative recombination centers. The 1550-nm emission has been obtained with InAs/GaAs QDs grown on metamorphic substrates, but poor reliability and repeatability remain as the severe issues for such technique [34]. In our previous work, the asymmetric InAs/GaAs QDs working at 1550 nm were fabricated, by which a mode-locked Er-doped glass oscillator has been achieved with 2 ps pulse width [24]. And recently, a 1550 nm QD-SESAM with InGaAs capped InAs/GaAs structure was fabricated, with which a dual-wavelength passively Q-switched erbium-doped fiber (EDF) laser has been achieved [35]. However, the performances of the obtained lasers were limited due to the small modulation depth of 0.4% of these QD-SESAMs. Therefore, it is highly desirable to explore new techniques to optimize the 1550 nm InAs/GaAs QD structures for the aim of enhancing the modulation depth of such QD-SESAMs.

In this work, we have grown different InAs/GaAs QD structures designed for SESAMs working at a 1550-nm range, with InGaAs alloy CLs and InGaAs short-period superlattice (SSL) CLs, respectively, and thoroughly investigated their optical properties. Photoluminescence (PL) spectroscopy characterization reveals very weak light emission at room temperature (RT) at the wavelength around 1550 nm, which cannot be observed at the lower temperature than 250 K. This phenomenon is in remarkable contrast to the well-known temperature-dependent behaviors of QD systems, namely, the PL intensity is stronger at lower temperatures, which gets very weak or even not observable at RT due to thermal excitation of the confined carriers in QDs. The abnormal phenomena observed in the 1550-nm InAs/GaAs QDs can be ascribed to the weak carrier relaxation process from CL to QDs, which can be significantly reduced by growing a SSL CL for the QDs. The SSL structures provide abundant phonon modes of large vibrational densities of states, which effectively boost the carrier relaxation from the CLs to the QDs. Therefore, a 10-time stronger 1550-nm emission than the non-SSL capped QDs is observed. The superior carrier dynamics in the 1550-nm QDs endows the QD-SESAMs with highly saturable absorption performance, manifested as a very small saturation intensity of 13.7 MW/cm^2^ and a larger nonlinear modulation depth of 1.6 % which is 4 times to the value reported in [24, 35]. Benefitting from the high performance of the QD-SESAM with SSL CLs, we have successfully constructed an EDF laser and achieved the stable mode–locked lasing at 1556 nm, with the pulse duration of 920 fs.

## Methods

### MBE growth of the InAs/GaAs QDs

**Fig. 1:**
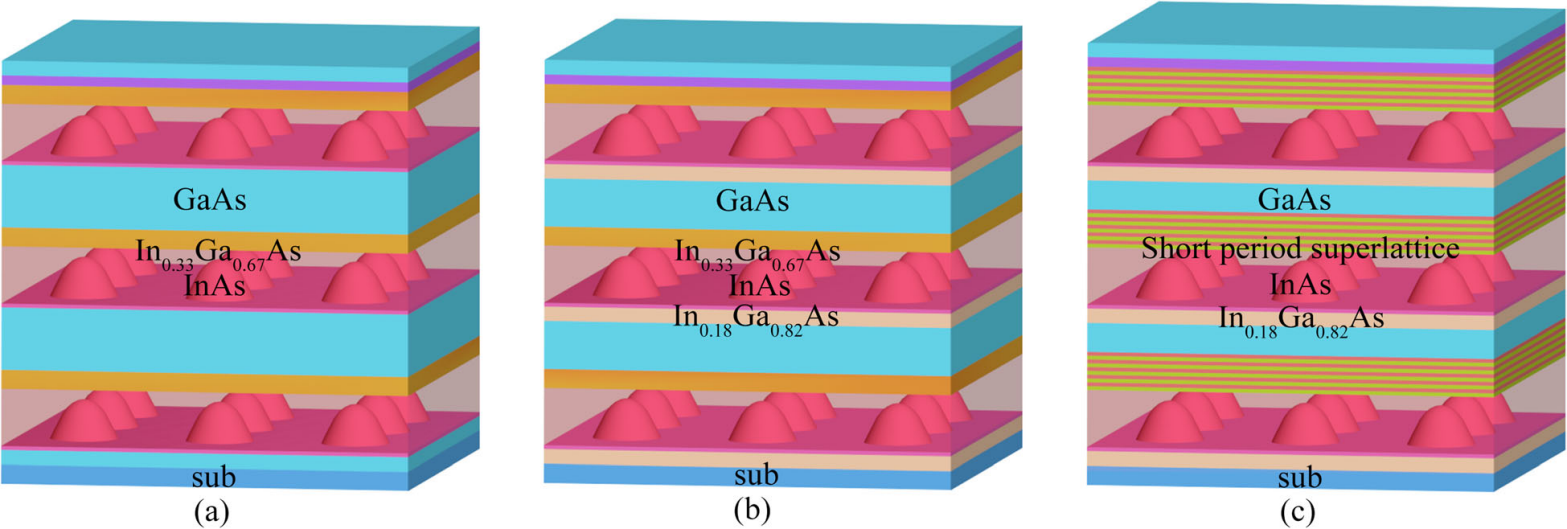
Schematic diagrams of QD structures. Schematic diagrams of three test structures of **a** sample 1, **b** sample 2, and **c** sample 3, respectively

Three InAs/GaAs QD structures were grown with the technique of molecular beam epitaxy (MBE). All the samples contain three periods of dot layers, each of which is self-assembled from 2.9 monolayers (MLs) InAs. As shown in Fig. 1, in samples 1 and 2, the InAs QDs were grown on GaAs and a 1-nm In_0.18_Ga_0.82_As buffer layer (BL), respectively, and all capped with a 6-nm-thick In_0.33_Ga_0.67_As layer. For sample 3, the 2.9 MLs InAs QDs were also grown on a 1-nm-thick In_0.18_Ga_0.82_As BL but capped with a 10-nm-thick SSL consisting of 5 periods of In_0.20_Ga_0.80_As (1 nm) and In_0.30_Ga_0.70_As (1 nm) layers. The growth temperature and the growth rate of InAs QDs were 510 °C and 0.01 ML/s, respectively. The QD-SESAMs were fabricated by growing one dot layer structure on a bottom Distributed Bragg Reflector (DBR) which contains 31 pairs of un-doped GaAs (115 nm) and Al_0.98_Ga_0.02_As (134 nm) layers. The growth temperatures for GaAs and InGaAs were 565 and 530 °C, respectively.

### Characterization Methods

PL measurements were performed at the varied temperatures range from 11 to 300 K with a 532-nm solid-state laser. The crystallography structures of these QD samples were characterized with high-resolution X-ray diffraction using the Cu Kα emission line. The morphologies of the QD structures were examined with the technique of atomic force microscope (AFM) in ambient conditions under a noncontact mode on a Nanoscope Dimension^TM^ 3100 SPM AFM system. Transmission electron microscopy (TEM) images were obtained on a 200 KeV JEOL-2010 microscope.

### Results and Discussion

**Fig. 2:**
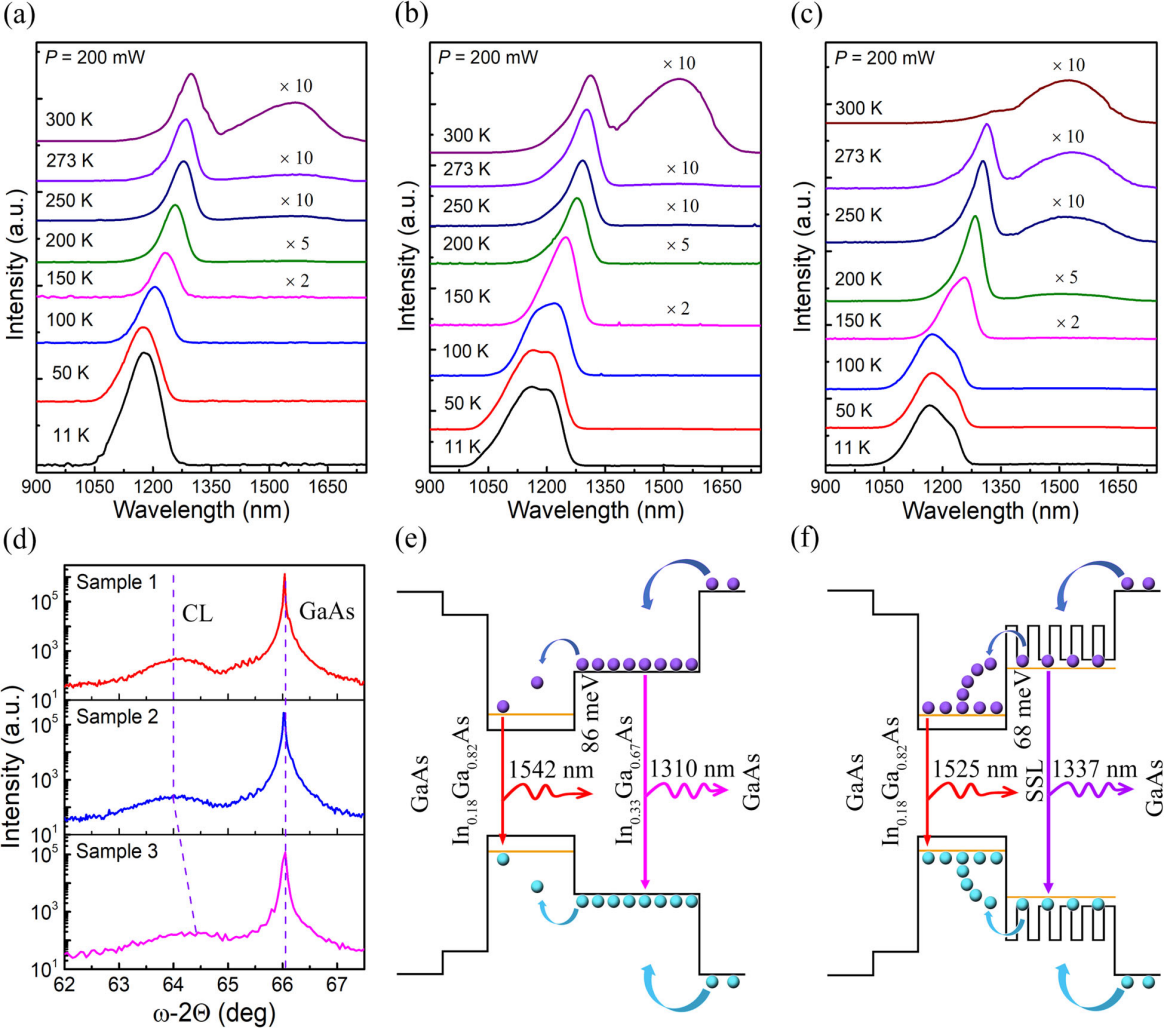
Material characterization and schematic band diagram. PL spectra measured at 11-300 K from **a** sample 1, **b** sample 2, and **c** sample 3, respectively. **d** High-resolution ω/2θ scans showing the GaAs (008) substrate peak and CL diffraction pattern for samples 1, 2, and 3, respectively. Schematic band diagram for **e** sample 2 and **f** sample 3, respectively

Fig. 2a, b, and c present the temperature-dependent PL spectra of samples 1, 2, and 3, respectively, acquired at the temperatures ranging from 11 to 300 K with the excitation power of 200 mW. It reveals two characteristic PL peaks, a narrow peak located at the short wavelength region and a broad one at the long wavelength. The narrow peak positioned at around 1170 nm at 11 K and about 1280 nm at 300 K originates from the luminescence from the CLs, while the broad peak at about 1550 nm at 300 K is ascribed to the QD emission. As shown in Fig. 2a, at the lower temperatures, only the CL emission can been observed, and the emission at around 1550 nm from the InAs QDs starts to appear when the temperature increases up to 250 K and gets gradually stronger with further increased temperature. The similar behavior is also observed with sample 2 as shown in Fig. 2b. Generally, for InAs/GaAs QD structures designed for shorter wavelength emission (e.g., 1300 nm), the emission from QDs dominates the PL spectra at low temperatures, and the emission from CLs or wetting layers hardly can be observed. This is because of the lower energy levels of the QD structures and reduced thermal escape of carriers from the QDs at low temperatures [36]. With the increase of the temperature, the emission intensity of QDs decreases gradually due to the enhanced thermal escape of carriers from QDs. In remarkable contrast to the InAs/GaAs QDs designed for 1310-nm applications, our samples for 1550 nm exhibit completely opposite temperature-dependent light emission behaviors, indicating distinguished carrier dynamics in this new QD system. As depicted in Fig. 2e, the bandgap of the QDs is much narrower than that of the CLs and the lowest energy levels for electrons and holes are all in the QD structure, and it is therefore expected that the photo-generated carriers may preferably reside in the QDs after relaxing their excessive energies. However, the observed PL result is that the CL emission dominates the PL and the QD emission is invisible at the temperature lower than 250 K, which reveals that, at low temperatures, the photo-generated carriers are dominantly confined in the CLs rather than in the QDs. This fact can be explained by the severe-carrier relaxation blocking effects that there are too few phonons participating in the carrier scattering process, resulting in the low relaxation efficiency of the carriers from the CLs to QDs. With the increase of temperature, more phonon populations are thermally excited and the interaction of phonons with photo-generated carriers is gradually enhanced, leading to more carriers scattered from CLs to the QDs. The strongest PL intensity of the QD emission at 1550 nm appearing at RT indicates that the energy relaxation process of the carriers from CLs to QDs dominates the thermal escape process from QDs to CLs.

The PL characterizations of samples 1 and 2 reveal that, in the InAs/GaAs QD system designed for 1550-nm applications, an inefficient carrier relaxation characteristic exists and larger phonon density favors the carrier relaxation down to QDs. Essentially, the weak carrier relaxation process is rooted in the electronic band structures determined by the CL. The QD materials in which their band offsets are much larger than the longitudinal optical (LO) phonon energies of the CLs and the carriers in the CLs have to relax to the QD levels via emitting multiple phonons rather than just a single one. The weak carrier relaxation process cannot be eliminated in the InAs/GaAs QDs for 1550 nm because of the much larger band offset in the CL and QD band structure, but we can modify the multiple phonon scattering process via adjusting the electronic band structures as well as the phonon band structures. To achieve this goal for enhancing the carrier relaxation in the 1550-nm QDs, we have employed a (In_0.20_Ga_0.80_As/In_0.30_Ga_0.70_As)_5_ SSL structure as the CLs to replace the InGaAs CLs. The SSL CLs are expected to provide more phonon vibrational modes and much larger phonon densities due to the Brillouin zone folding effects in SSL [37]. As shown in Fig. 1c, sample 3 was grown with the same structure as sample 2 except the usage of five periods of 10-nm-thick In_0.20_Ga_0.80_As/In_0.30_Ga_0.70_As SSLs as the CLs. Figure 2d shows the obtained XRD patterns for samples 1, 2, and 3. All the samples exhibit a strong peak at 66.1°, which can be assigned to the diffraction from the (008) planes of cubic GaAs. Clear satellite peaks resulting from the 6-nm-thick In_0.33_Ga_0.67_As CL structure are observed at around 64.0° for samples 1 and 2. Further inspection reveals that In_0.20_Ga_0.80_As/In_0.30_Ga_0.70_As SSL in sample 3 exhibits a satellite peak at around 64.4°, and the shift towards larger degrees with respect to that of In_0.33_Ga_0.67_As CLs suggests a decrease of the average In content [38, 39]. To understand the effect of SSL CLs on the optical properties of the InAs/GaAs QDs, temperature-dependence PL spectra for sample 3 are also measured as shown in Fig. 2c. Similar to sample 1 and 2, no obvious PL emission at 1550 nm from the InAs/GaAs QDs can be observed at temperatures lower than 200 K and the emission gets gradually intense with increased higher temperatures. It’s worth noting that the QD emission peak at 1550 nm in sample 3 emerges at a much lower temperature of 200 K (around 250 K for samples 1 and 2). Its relative intensity with respect to the CL emission at RT is much higher than samples 1 and 2, and its PL intensity is about 10 times stronger than sample 2. These results indicate the SSL CLs greatly boost the carrier relaxation from the CLs down to the QDs, resulting in much enhanced radiative recombination in the QDs. The reason responsible for the enhanced carrier relaxation from the CLs to the QDs lies in high-quality SSL CLs with the decreased indium content. This effectively modulates the carrier relaxation behaviors and enhances the capture of carriers by the QDs.

To make further insight into the multi-phonon-facilitated carriers scattering process, the band structures of the InAs/GaAs QD system with different types of CLs are compared. For the aim of simplicity, the energy difference between the CL and QD bandgaps can be estimated as the difference in their PL peak energies. As shown in Fig. 2e and f, the bandgap differences in sample 2 and 3 between the CL and the InAs QDs at 300 K are determined as 143 and 114 meV, respectively, according to the PL measurements. With the assumption that the band offsets are approximately 60 % of energetic differences between conduction bands of CL and QDs [40], electrons must relax 86 and 68 meV for sample 2 and sample 3, respectively, to be scattered from the energy levels of the capping layers to the lowest energy levels of the InAs QDs. The phonon energies of the LO and longitudinal acoustic (LA) modes in the InGaAs alloys are 34 and 9 meV [40, 41]. For the multiple phonon scattering process, the combination of 2 LO phonons in sample 3 can fulfill the scattering of an electron from the CLs to QDs while 2 LO phonons plus 1 LO or 2 LA phonons are required for sample 2. It has been demonstrated that electron relaxation rate is severely reduced when more phonon modes are involved in a multiple phonon scattering process [42–45]. Therefore, the electron relaxation rate in sample 3 is larger than that in sample 2, which accounts for the much enhanced PL intensity of the QDs in sample 3. Actually, the decreased In content in the SSL CLs and the weakened phonon bottleneck effect in the carrier relaxation process are the main reasons for the enhanced PL intensity of the QDs in sample 3.

**Fig. 3:**
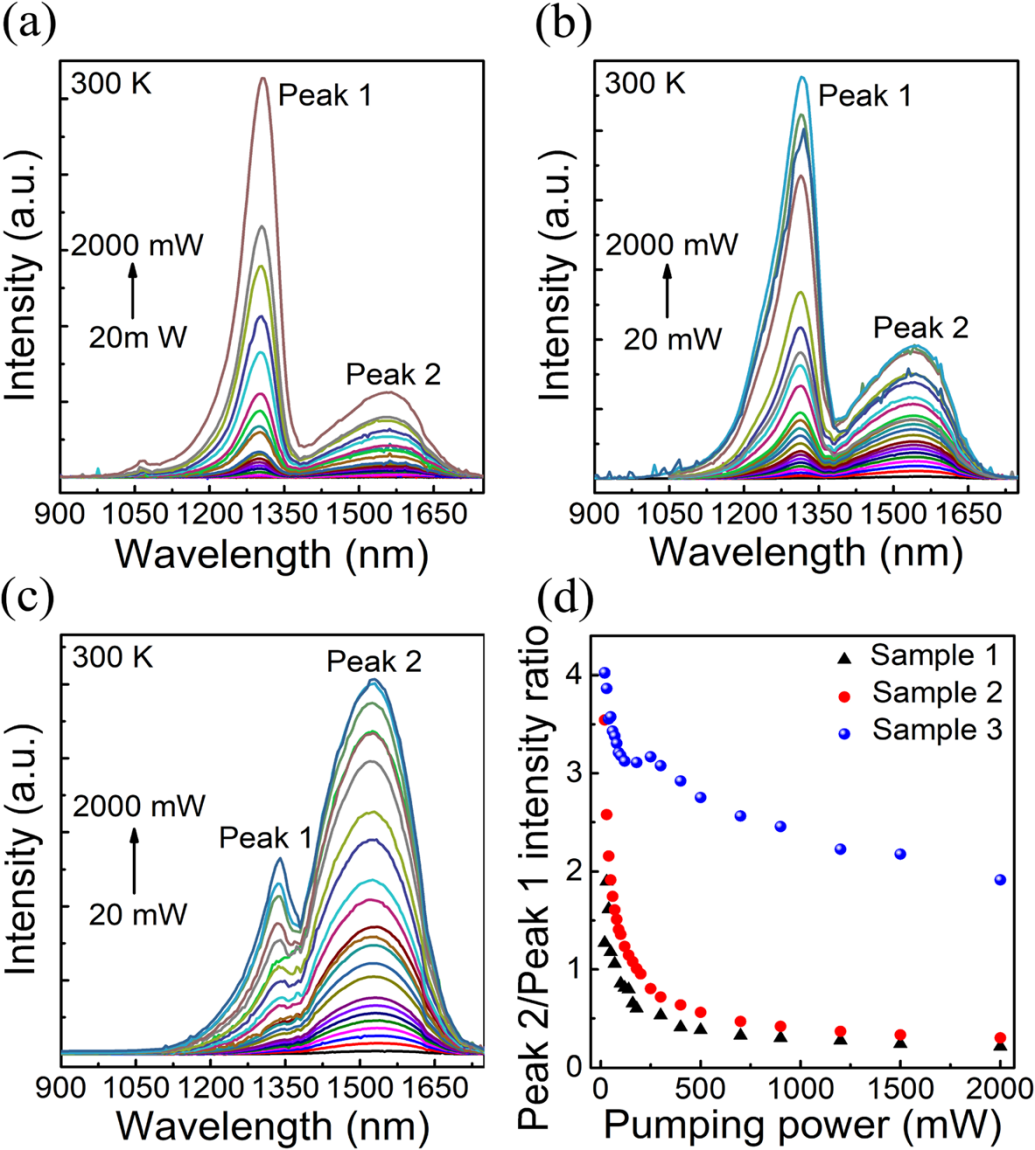
Power dependent PL measurements. Room temperature power-dependent PL spectra measured at 20–2000 mW from **a** sample 1, **b** sample 2, and **c** sample 3, respectively. **d** Intensity radio of Peak 2/Peak 1 versus pumping power in samples 1, 2, and 3, respectively.

To further verify the enhanced carrier relaxation effect caused by the SSL CLs, excitation power–dependent PL spectra were acquired at 300 K. As shown in Fig. 3a, b, and c, the PL intensity of the CL (Peak 1) and InAs QD (Peak 2) peaks increases gradually with the increased excitation power, and no obvious shift of the peak positions can be observed. It is clearly observed that the intensity of Peak 1 is much stronger than that of Peak 2 in sample 1 and 2 as shown in Fig. 3a and b at the higher excitation power, while sample 3 exhibits a much stronger QD emission in all measured excitation power range. The PL intensity ratios of Peak 2 and Peak 1 of these samples as a function of excitation power were summarized in Fig. 3d. At the excitation power of 2000 mW, the PL intensity ratios of Peak 2 and Peak 1 are found to be 0.21 and 0.29 corresponding to sample 1 and 2, respectively, as shown in Fig. 3d. It indicates that a lot of carriers recombine in InGaAs CL and the carrier relaxation from capping layer to InAs QDs is severely hindered due to the inefficient carrier relaxation rate. Compared with sample 1, the layer intensity ratio of Peak 2 to Peak 1 in sample 2 may be attributed to the higher dot density achieved by more nucleation centers caused by the In_0.18_Ga_0.82_As buffer layer [24]. The intensity of Peak 2 in sample 3 is about 2.1 times stronger than that of Peak 1 at the excitation power of 2000 mW, indicating much enhanced carrier relaxation efficiency in the SSL capped InAs QDs. In addition, it is found that although the average In content is around 25 % in the SSL capping layer that is smaller than 33 % in the CLs of samples 1 and 2, the emission wavelength of peak 1 (at ~1337 nm) in sample 3 is slightly longer than that (at ~ 1310 nm) for samples 1 and 2. We believed that the main reason responsible for the results is the reduced quantum confinement effect in the much thicker (10 nm) SSL layer compared with the 6-nm InGaAs capping layer.

**Fig. 4:**
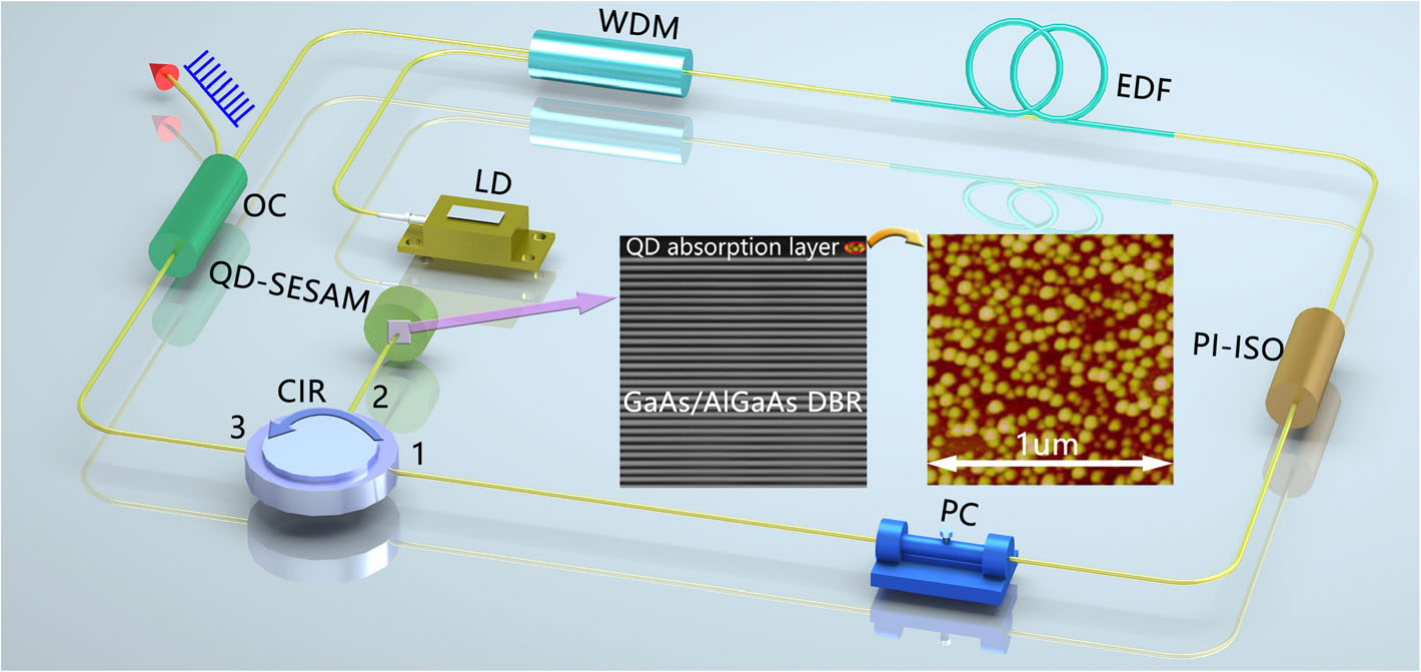
Experimental setup of mode-locked fiber laser with 1550-nm QD-SESAM. Inset: cross-sectional TEM image of the QD-SESAM and 1 × 1 μm^2^ AFM image of the 1550-nm QDs

Based on the good optical properties obtained in the SSL capped InAs/GaAs QDs, we further demonstrate its application as a QD-SESAM for femtosecond pulse generation. The 1550 nm SSL capped InAs/GaAs QD-SESAM consists of one layer of SSL capped InAs/GaAs QDs as the absorption layer and a bottom DBR mirror made of 31 periods of un-doped GaAs (115 nm) and Al_0.98_Ga_0.02_As (134 nm) layers. The detailed structure of the QD-SESAM is illustrated by the cross-sectional TEM image as shown in Fig. 4. The average dot density of the QDs in the absorption layer is estimated to be 4.4 × 10^10^ cm^-2^, and the average height and lateral size of the dot are 7.5 and 40 nm respectively as seen in the AFM image in Fig. 4. The SESAM is characterized with a typical balanced twin-detector setup [46] and a saturation intensity of 13.7 MW/cm^2^ and a nonlinear modulation depth of 1.6 % are achieved. As depicted in Fig. 4, with the QD-SESAM inserted in the EDF laser cavity, we have constructed a passively mode-locked laser. With a standard 23.75-m single-mode fiber and a 0.75-m EDF as the gain medium, the cavity obtained is 24.5 m in length. A semiconductor DFB laser diode (LD) emitting at 980 nm serves as the pump source, and a 980/1550 nm wavelength division multiplexer (WDM) is used to couple the pump energy into the fiber laser cavity. A polarization-independent isolator (PI-ISO) and a polarization controller (PC) are used to ensure one-way transmission of the light and optimizing mode-locking state in the cavity, respectively. Port 1 of a 1550-nm optical circulator (CIR) is connected to the PC, Port 2 is linked to the QD-SESAM, and Port 3 of this CIR is connected to the 10/90 output coupler (OC) (10% output and 90% input).

**Fig. 5:**
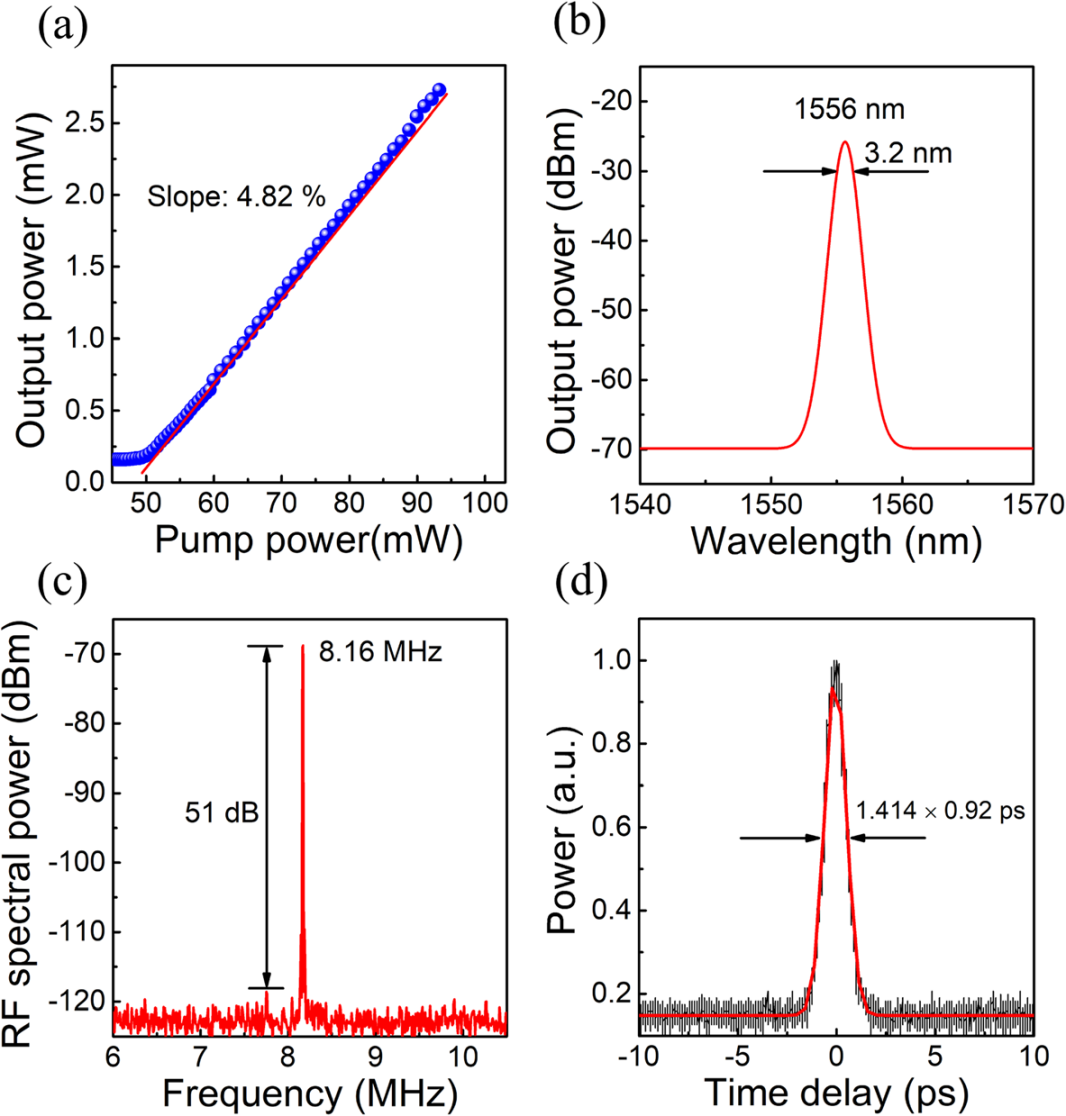
Characteristics of mode-locked the developed fiber laser. **a** Output power versus pump power. **b** Output optical spectra. **c** RF spectrum of the mode-locked fiber laser. **d** Autocorrelation trace

The mode-locking behavior can be achieved when the pump power is higher than 50 mW. As shown in Fig. 5a, the output power of this mode-locked laser linearly increases with the increased pump power and the slope efficiency is about 4.82% determined by the linear fitting treatment. As presented in Fig. 5b, the typical spectrum of the conventional soliton with a 3-dB bandwidth of 3.2 nm was observed. The central wavelength is 1556 nm. The RF spectrum with a repetition rate of 8.16 MHz is shown in Fig. 5c, corresponding to the cavity length of 24.5 m. The signal-to-noise ratio is about 51 dB, indicating the great potential to achieve stable mode-locking operation with the SSL capping QD-SESAMs. Long-time stable mode-locking measurements were operated at the threshold pump power of 50 mW, and over 1 week of stable continuous operation was achieved. Fig. 5d is the autocorrelation trace fitted with a Gaussian fitting profile, which illustrates the real pulse duration of approximately 920 fs. For comparison, with the QD-SESAM based on the structure as in sample 2 exhibiting a saturation intensity of 15.7 MW/cm^2^ and a nonlinear modulation depth of 0.4 %, and the mode-locked laser generates pulses of 2.7 ps wide [47]. The much reduced pulse duration achieved with QD-SESAM based SSL capped QDs can be ascribed to the increased modulation depth, and we believed that the enhanced carrier relaxation efficiency induced by SSL capping layers accounts for the decreased saturation intensity. Moreover, five other SSL capped QD-SESAMs have been selected to construct the mode-locked fiber lasers, and all the mode-locked lasers have exhibited long-term stability, by which the high repeatability and reliability of the SESAMs are demonstrated.

## Conclusions

In conclusion, InAs/GaAs QDs designed for 1550 nm applications were grown by the MBE technique with layers of InGaAs alloy and SSL, respectively, as the capping layers for QDs. With temperature-dependent and power-dependent PL spectroscopy characterization, it is revealed that the conduction band offset of CL and QD structures is modified from 86 meV down to 68 meV by changing the In_0.33_Ga_0.67_As alloy CL to a (In_0.20_Ga_0.80_As/In_0.30_Ga_0.70_As)_5_ SSL CL, and more efficient multiple-phonon-involved carrier scattering is therefore achieved, which leads to more carriers radiatively recombining in the QD structure and the resultant significantly improved emission at 1550 nm. The QD-SESAM grown with the SSL capped InAs/GaAs QDs exhibits a much enhanced saturation intensity of 13.7 MW/cm^2^ and a nonlinear modulation depth of 1.6%, and a pulse duration of 920 fs is achieved in a mode-locked fiber laser operating at 1556 nm constructed with the QD-SESAM. The developed QD-SESAM with the SSL design as the CLs for QDs will pave a new way towards high-performance ultrafast lasers.

## Data Availability

The datasets generated and/or analyzed during the current study are fully available without restriction from the corresponding author on reasonable request.

## Abbreviations

2D: Two-dimensional
AFM: Atomic force microscope
BL: Buffer layer
CIR: Circulator
CLs: Capping layers
DBR: Distributed Bragg Reflector
EDF: Erbium-doped fiber
LA: Longitudinal acoustic
LD: Laser diode
LO: Longitudinal optical
MBE: Molecular beam epitaxy
MLs: Monolayers
OC: Output coupler
PC: Polarization controller
PI-ISO: Polarization-independent isolator
PL: Photoluminescence
QDs: Quantum dots
QW: Quantum well
RT: Temperature
SA: Saturable absorber
SESAMs: Semiconductor saturable absorber mirrors
SSL: Short-period superlattice
TEM: Transmission electron microscopy
WDM: Wavelength division multiplexer
